# Stratifying the stratifiers of triple negative breast cancer

**DOI:** 10.18632/oncotarget.27455

**Published:** 2020-01-28

**Authors:** Dong-Yu Wang, Zhe Jiang, Eldad Zacksenhaus

**Keywords:** triple negative breast cancer, molecular stratification, prognostication, microRNA, immunomodulation

Triple-negative breast cancer (TNBC) is a highly heterogeneous disease that can be molecularly stratified into at least six different subtypes. Whether each subtype is homogeneous or can be further stratified is of great clinical interest. We have recently reported that three of these six TNBC subtypes, basal-like 1 (BL1), basal-like 2 (BL2) and immunomodulatory (IM), can be stratified on the basis of PTEN status plus expression of five microRNAs or RHOA pathway activity, AKT1 amplification/expression, and PD-1 expression, respectively, with dramatic effects on overall survival. This editorial describes the research that has led to this new stratification of TNBC subtypes and its implications for therapy.

## Breast cancer heterogeneity and triple negative breast cancer subtypes

Breast Cancer (BC) is clinically classified as Estrogen-receptor positive (ER^+^), HER2-positive (HER2^+^) and triple negative (TNBC) subtypes, the latter of which express low levels of ER, progesterone-receptor or HER2. TNBC patients constitute 10–15% of all cases in the general population, and up to 50% in women of African origin. Using gene expression based classification, Lehmann et al. sub-divided TNBCs into six subtypes: basal-like 1 (BL1), basal-like 2 (BL2), mesenchymal (M), mesenchymal stem-like (MSL), immunomodulatory (IM), and luminal androgen receptor (LAR), as well as an unspecified group (UNS) [1]. The overall survival (OS) of each subtype shows a wide distribution with some patients succumbing to their disease within a few years while others surviving for over 5–10 years from the time of diagnosis. Whether this heterogeneity is stochastic or driven by differences in oncogenic alterations in each subtype is a critical question that can guide treatment.

## microRNAs' impact on tumorigenesis of Pten-deficient mammary tumors – from mouse models to human TNBC patients

We began this study by analyzing tumor initiating cells (TICs) in mouse models of Pten-deficient mammary tumor cells. Deletion of the tumor suppressor Pten via two different CRE drivers (MMTV-Cre and WAP-CRE) led to histologically heterogeneous tumors [2]. Remarkably, under conditions in which tumor cells from other mouse models (Rb-loss, p53-loss, HER2+, WNT+) sprouted secondary lesions following orthotopic transplantation [2–5], Pten-deficient tumors failed to engraft into immune-competent or even immune-deficient recipient mice [6]. Systematic screening of over 100 WAP-Cre:Pten^f/f^ tumors identified a small fraction (6.8%) of transplantable tumors that exhibited distinct histology, molecular classification, signaling pathways, chromosomal aberrations and mutational landscapes, as well as reduced expression of microRNA-143/145 [6]. Stable knockdown of miR-143/145 increased tumorigenic potential, and enhanced RAS signaling and sensitivity to MEK inhibition. In human TNBC, miR145-deficiency correlated with elevated RAS pathway activity, and patients with combined PTEN-low/miR-145 low expression exhibited poor clinical outcome [6]. Interestingly, comparison of histologically and molecularly similar mammary tumors driven by Pten-loss versus activating Pik3ca mutation revealed reduced EGFR signaling in the former [7].

**Figure 1:**
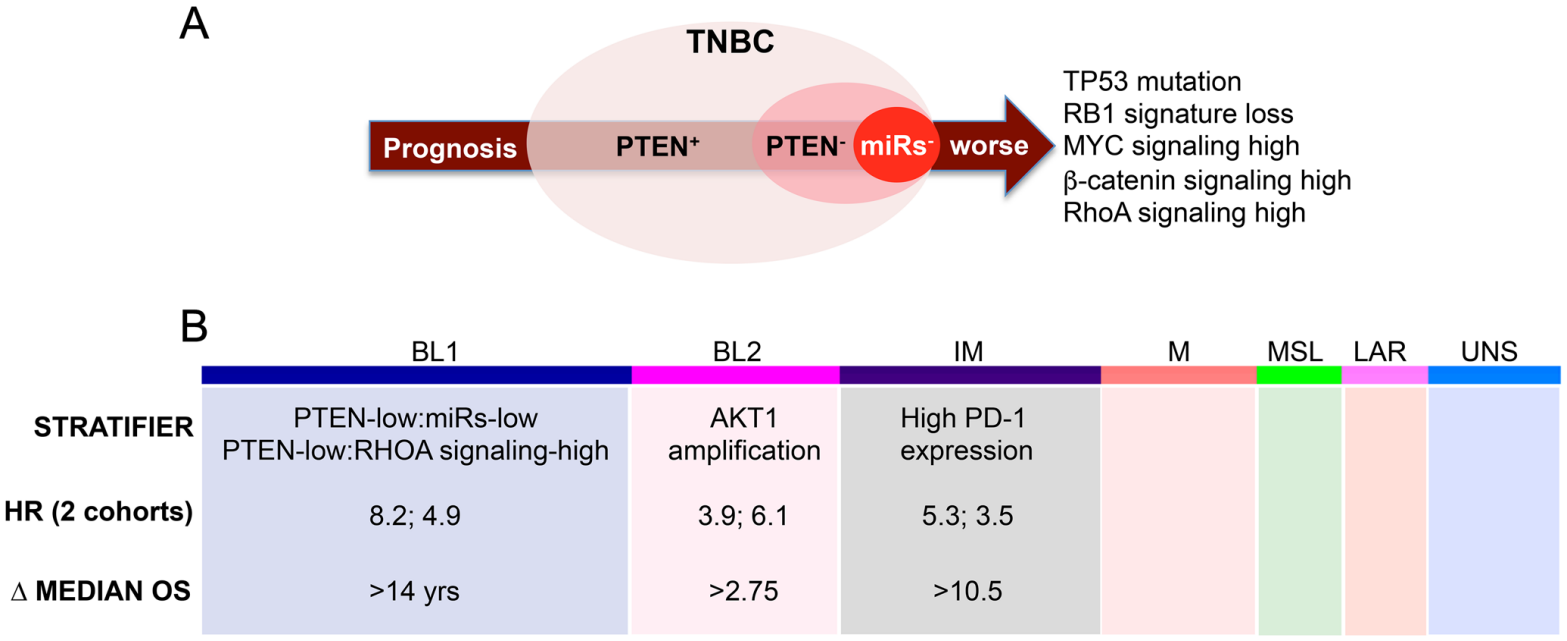
Startification within TNBC subtypes. (**A**) PTEN-deficient TNBCs with low expression of 4 of 5 microRNAs (see text) have exceptionally poor clinical outcome. These tumors exhibit TP53 mutation, RB1 signature loss, and high MYC, WNT and RHOA signaling. (**B**) TNBC subtypes and stratification within BL1, BL2 and IM. The PTEN-low:miRs-low subgroup (A) clusters with BL1. BL2 and IM can be stratified on the basis of AKT1 amplification/high mRNA expression, or high PD-1 mRNA expression, respectively. Δ median OS – differences in median overall survival of stratifier-positive vs –negative patients. HR – hazard ratio.

These results raised the question of whether other or additional microRNAs may cooperate with PTEN loss to induce TNBCs with poor clinical outcome. To assess this possibility, we systematically searched for microRNAs with expression patterns that correlated with PTEN mRNA levels, and then determined the prognostic power of each PTEN-miRNA pair. Strikingly, in TNBC, reduced expression of hsa-miR-145 as well as hsa-miR-4324, hsa-miR-125b, hsa-miR-381 and has-miR136 correlated with low PTEN expression [8]. Combined loss of PTEN together with 4 of 5 of these miRNAs identified a subgroup of patients with exceedingly poor clinical outcome with hazard ratios (HRs) of 3.91 (*P* < 0.0001) and 4.42 (*P* = 0.0003) in two independent clinical cohorts. Genomic analysis of the PTEN-low/miRs-low subgroup revealed TP53 mutation (rater than deletion or HDM2 amplification), RB1-loss signature and high PI3K, MYC and β-catenin signaling (Figure 1A).

## Stratifying the stratifiers and clinical implications

We next asked whether these aggressive PTEN-low/miRs-low TNBC lesions originated from a single or multiple different TNBC subtypes. Strikingly, nearly all PTEN-low/miRs-low TNBC samples clustered as basal-like 1 (BL1) TNBC [9]. These BL1 lesions showed high RHOA signaling. PTEN-low/RHOA-signalling-high BL1 tumors displayed the worst prognosis with HRs of 8.2 (*P* = 0.0009) and 4.87 (*P* = 0.033) in the two different cohorts. The difference in median OS between PTEN-low/RhoA-signalling-high and –low BL1 was over 14 years. The two groups of patients are therefore quite distinct and should be treated with different priorities and therapeutic regimens (Figure 1B).

These observations prompted a search for factors that can stratify other TNBC subtypes. Gene expression profiles, mutational landscapes, chromosomal gains/losses and signaling pathway activities identified chromosome 14q32.3 as a region of gain in multiple BL2 TNBC lesions. High copy number alteration (CNA) and mRNA expression of AKT1 but not other genes on the amplicon predicted poor clinical outcome in BL2 with HRs of 3.9 (*P* = 0.02) and 6.1 (*P* = 0.0032), and a survival difference of 2.75 years between high and low AKT1 CNA groups. In addition, BL2 TNBCs feature high E2F2 and TGF-β signalling as well as high CXCL8 expression, which may be therapeutically targetable (Figure 1B).

For the IM subtype, mRNA expression of programmed cell death 1 (PD1) was predictive of poor prognosis with HRs of 5.3 (*P* = 0.01) and 3.5 (*P* < 0.004), and a difference of over 10.5 years in OS between high and low PD-1 expressing tumors. A recent phase III clinical trial of advanced and metastatic TNBC revealed moderate response to anti-PD-L1 therapy in combination with cytotoxic chemotherapy (paxlitaxel), but patients still succumbed to their disease [10]. The identification of markers that can predict response is of paramount importance. Our results suggest that IM TNBC patients with high PD-1 expression may be particularly sensitive to anti-PD-1 therapy. In addition, IM TNBCs express high IFNα and IFNγ signalling and CTLA4 mRNA expression that may also be targeted for therapy (Figure 1B).

We were so far unable to identify oncogenic alterations or markers that can stratify the other TNBC subtypes, though MSL TNBC samples exhibited high EGFR signalling. Additional analysis using other classifiers such as proteomics and metabolomics may uncover mechanisms to segregate the other TNBC subtypes: M, MSL, and LAR. However, for BL1, BL2 and IM, our results clearly demonstrate that OS is not stochastic but driven by inherent oncogenic heterogeneity that impacts tumor progression.

This intrinsic heterogeneity can be exploited not only to prioritize patients for therapy but also to devise precision medicine. Thus, BL1 TNBCs are predicted to be sensitive to drugs that target PTEN loss such as PI3K/AKT inhibitors, CHK1/CDC25/WEE1/Aurora kinase pathway that target RB loss, and drugs that target TP53 mutation, MYC and WNT signaling; BL2 lesions are predicted to be sensitive to AKT inhibitors; whereas IM tumors to anti-PD-1 therapy (Figure 1B). Tumors from high-risk patients of each subtype may also be further interrogated for additional oncogenic addictions and predicted drug sensitivity or through high content pharmacological and genetic screens for druggable targets or synthetic lethal interactions.

In conclusion, BL1, BL2 and IM TNBC, and possibly additional subtypes, can be stratified into patients with extremely poor prognosis and others with much better outcome. The former tumors should be identified and prioritized for precision medicine based on their oncogenic landscapes and vulnerabilities. Although highly significant results were obtained with two large and independent clinical cohorts, analysis of additional patients/cohorts will further increase confidence in these new stratifications. The development of a small number of markers for immunohistochemistry or mRNA probes for NanoString technology to replace multi-gene classifiers and the Lehmann’s based TNBC subtyping would streamline the identification and treatment of high-risk patients of each TNBC subtype.
